# *N*-(Adamantan-1-yl)-1,2,3,4-tetra­hydro­iso­quinoline-2-carbo­thio­amide

**DOI:** 10.1107/S1600536813031516

**Published:** 2013-11-23

**Authors:** Ali A. El-Emam, Ebtehal S. Al-Abdullah, Hanaa M. Al-Tuwaijri, C. S. Chidan Kumar, Hoong-Kun Fun

**Affiliations:** a Department of Pharmaceutical Chemistry, College of Pharmacy, King Saud University, PO Box 2457, Riaydh 11451, Saudi Arabia; b X-ray Crystallography Unit, School of Physics, Universiti Sains Malaysia, 11800 USM, Penang, Malaysia

## Abstract

In the title compound, C_20_H_26_N_2_S, the N-containing six-membered ring adopts a boat conformation and the dihedral angle between the thio­carbamide group and the benzene ring is 49.67 (9)°. An intra­molecular C—H⋯S hydrogen bond generates an *S*(6) ring motif. The N—H group is sterically hindered and there are no significant inter­molecular inter­actions beyond van der Waals contacts.

## Related literature

For related structures and biological background, see: Al-Abdullah *et al.* (2012 ▶); El-Emam *et al.* (2012 ▶); Al-Tamimi *et al.* (2013 ▶).
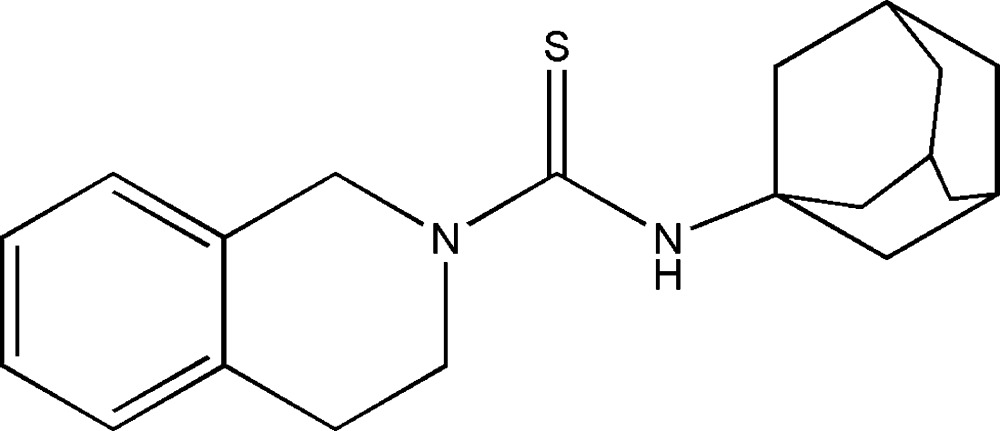


## Experimental

### 

#### Crystal data

C_20_H_26_N_2_S
*M* *_r_* = 326.49
Monoclinic, 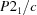

*a* = 19.1707 (5) Å
*b* = 6.4106 (2) Å
*c* = 14.2838 (3) Å
β = 103.366 (2)°
*V* = 1707.87 (8) Å^3^
*Z* = 4
Cu *K*α radiation
μ = 1.67 mm^−1^
*T* = 296 K
0.81 × 0.13 × 0.05 mm

#### Data collection

Bruker APEXII CCD diffractometer
Absorption correction: multi-scan (*SADABS*; Bruker, 2009 ▶) *T*_min_ = 0.345, *T*_max_ = 0.921
10894 measured reflections
2831 independent reflections
2351 reflections with *I* > 2σ(*I*)
*R*_int_ = 0.040

#### Refinement

*R*[*F* ^2^ > 2σ(*F* ^2^)] = 0.041
*wR*(*F* ^2^) = 0.114
*S* = 1.07
2831 reflections
212 parameters
H atoms treated by a mixture of independent and constrained refinement
Δρ_max_ = 0.16 e Å^−3^
Δρ_min_ = −0.20 e Å^−3^

### 

Data collection: *APEX2* (Bruker, 2009 ▶); cell refinement: *SAINT* (Bruker, 2009 ▶); data reduction: *SAINT*; program(s) used to solve structure: *SHELXTL* (Sheldrick, 2008 ▶); program(s) used to refine structure: *SHELXTL*; molecular graphics: *SHELXTL*; software used to prepare material for publication: *SHELXTL* and *PLATON* (Spek, 2009 ▶).

## Supplementary Material

Crystal structure: contains datablock(s) global, I. DOI: 10.1107/S1600536813031516/hb7163sup1.cif

Structure factors: contains datablock(s) I. DOI: 10.1107/S1600536813031516/hb7163Isup2.hkl

Supplementary material file. DOI: 10.1107/S1600536813031516/hb7163Isup3.cml

Additional supplementary materials:  crystallographic information; 3D view; checkCIF report

## Figures and Tables

**Table 1:** Hydrogen-bond geometry (Å, °)

*D*—H⋯*A*	*D*—H	H⋯*A*	*D*⋯*A*	*D*—H⋯*A*
C16—H16*B*⋯S1	0.97	2.72	3.365 (2)	125

**Fig. 1.**
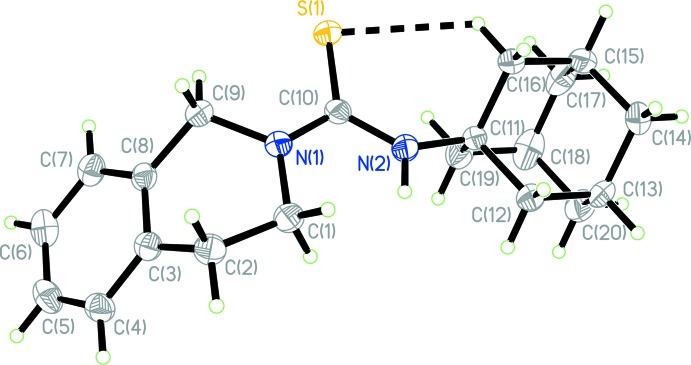
The molecular structure of the title compound with 30% probability displacement ellipsoids.

## supplementary crystallographic information

### 1. Comment

In continuation to our interest in the chemical and pharmacological properties of adamantane derivatives, (Al-Abdullah *et al.*, 2012; El-Emam *et al.*, 2012; Al-Tamimi *et al.*, 2013) we synthesized the title compound (I) as a potential bioactive agent and its crystal structure is described here.

In the crystal structure of the title compound (I), the N-containing six-membered (N1/C1—C3/C8/C9) ring, Fig. 1, adopts a boat conformation, puckering parameters: Q = 0.639 (2) Å, θ = 88.01 (18)°, and φ = 119.18 (18)° with a maximum deviation of 0.380 (2) Å at atom C2. An intramolecular C–H···S hydrogen bond form a six-membered ring, Fig. 1, generating an *S*(6) ring motif, In the crystal structure, no significant intermolecular hydrogen bonds are observed.

### 2. Experimental

A mixture of 387 mg 1-adamantylisothiocyanate (387 mg, 2 mmol) and 1,2,3,4-tetrahydroisoquinoline (266 mg, 2 mmol), in ethanol (15 ml), was heated under reflux for 2 h. On cooling, the precipitated crude product were filtered, washed with cold ethanol, dried, and crystallized from ethanol to yield 575 mg (88%) of the title compound (C_20_H_26_N_2_S), *m.p.*: 420–422 K. Colorless needles were obtained from the slow evaporation of a CHCl_3_:EtOH solution (1:1; 5 ml) at room temperature.

^1^H NMR (CDCl_3_, 500.13 MHz): δ 1.60–1.67 (m, 6H, Adamantane-H), 2.05 (s, 3H, Adamantane-H), 2.25–2.26 (m, 6H, Adamantane-H), 2.85 (t, 2H, J = 6.0 Hz, Isoquinoline-CH_2_), 3.81 (t, 2H, J = 6.0 Hz, Isoquinoline-CH_2_), 4.80 (s, 2H, Isoquinoline-CH_2_), 5.15 (s, 1H, NH), 7.08–7.15 (m, 4H, Ar—H). ^13^C NMR (CDCl_3_, 125.76 MHz): δ 29.68, 32.88, 35.56, 42.01 (Adamantane-C), 29.24, 48.12, 54.82, 126.49, 127.08, 128.33, 129.14, 133.43, 135.50 (Isoquinoline-C), 179.46 (C=S).

### 3. Refinement

All H atoms were positioned geometrically [C—H = 0.93, 0.97 or 0.98 Å] and refined using a riding model with *U*_iso_(H) = 1.2 *U*_eq_(C).

### Crystal data

**Table:**

C_20_H_26_N_2_S	*F*(000) = 704
*M**_r_* = 326.49	*D*_x_ = 1.270 Mg m^−^^3^
Monoclinic, *P*2_1_/*c*	Cu *K*α radiation, λ = 1.54178 Å
Hall symbol: -P 2ybc	Cell parameters from 2009 reflections
*a* = 19.1707 (5) Å	θ = 4.7–68.5°
*b* = 6.4106 (2) Å	µ = 1.67 mm^−^^1^
*c* = 14.2838 (3) Å	*T* = 296 K
β = 103.366 (2)°	Needle, colorless
*V* = 1707.87 (8) Å^3^	0.81 × 0.13 × 0.05 mm
*Z* = 4	

### Data collection

**Table:**

Bruker APEXII CCD diffractometer	2831 independent reflections
Radiation source: fine-focus sealed tube	2351 reflections with *I* > 2σ(*I*)
Graphite monochromator	*R*_int_ = 0.040
φ and ω scans	θ_max_ = 65.0°, θ_min_ = 4.7°
Absorption correction: multi-scan (*SADABS*; Bruker, 2009)	*h* = −22→22
*T*_min_ = 0.345, *T*_max_ = 0.921	*k* = −5→7
10894 measured reflections	*l* = −16→16

### Refinement

**Table:**

Refinement on *F*^2^	Primary atom site location: structure-invariant direct methods
Least-squares matrix: full	Secondary atom site location: difference Fourier map
*R*[*F*^2^ > 2σ(*F*^2^)] = 0.041	Hydrogen site location: inferred from neighbouring sites
*wR*(*F*^2^) = 0.114	H atoms treated by a mixture of independent and constrained refinement
*S* = 1.07	*w* = 1/[σ^2^(*F*_o_^2^) + (0.0467*P*)^2^ + 0.4078*P*] where *P* = (*F*_o_^2^ + 2*F*_c_^2^)/3
2831 reflections	(Δ/σ)_max_ = 0.001
212 parameters	Δρ_max_ = 0.16 e Å^−^^3^
0 restraints	Δρ_min_ = −0.20 e Å^−^^3^

### Special details

**Table:**

Geometry. All e.s.d.'s (except the e.s.d. in the dihedral angle between two l.s. planes) are estimated using the full covariance matrix. The cell e.s.d.'s are taken into account individually in the estimation of e.s.d.'s in distances, angles and torsion angles; correlations between e.s.d.'s in cell parameters are only used when they are defined by crystal symmetry. An approximate (isotropic) treatment of cell e.s.d.'s is used for estimating e.s.d.'s involving l.s. planes.
Refinement. Refinement of *F*^2^ against ALL reflections. The weighted *R*-factor *wR* and goodness of fit *S* are based on *F*^2^, conventional *R*-factors *R* are based on *F*, with *F* set to zero for negative *F*^2^. The threshold expression of *F*^2^ > σ(*F*^2^) is used only for calculating *R*-factors(gt) *etc*. and is not relevant to the choice of reflections for refinement. *R*-factors based on *F*^2^ are statistically about twice as large as those based on *F*, and *R*- factors based on ALL data will be even larger.

### Fractional atomic coordinates and isotropic or equivalent isotropic displacement parameters (Å^2^)

**Table:**

	*x*	*y*	*z*	*U*_iso_*/*U*_eq_	
S1	0.28539 (3)	0.53707 (8)	0.87853 (4)	0.04780 (18)	
N1	0.31695 (8)	0.8392 (2)	1.01049 (11)	0.0410 (4)	
N2	0.22585 (9)	0.9145 (3)	0.88193 (13)	0.0487 (4)	
C1	0.30653 (10)	1.0463 (3)	1.04991 (15)	0.0454 (5)	
H1A	0.2582	1.0555	1.0596	0.054*	
H1B	0.3118	1.1525	1.0037	0.054*	
C2	0.35954 (10)	1.0889 (3)	1.14462 (14)	0.0468 (5)	
H2A	0.3574	1.2352	1.1611	0.056*	
H2B	0.3466	1.0071	1.1952	0.056*	
C3	0.43444 (10)	1.0346 (3)	1.13817 (13)	0.0394 (4)	
C4	0.49318 (11)	1.1642 (3)	1.16802 (14)	0.0477 (5)	
H4A	0.4874	1.2961	1.1922	0.057*	
C5	0.56065 (11)	1.0963 (4)	1.16170 (15)	0.0541 (6)	
H5A	0.6002	1.1827	1.1819	0.065*	
C6	0.56903 (11)	0.9017 (4)	1.12565 (15)	0.0549 (6)	
H6A	0.6146	0.8556	1.1231	0.066*	
C7	0.51028 (10)	0.7734 (3)	1.09301 (13)	0.0485 (5)	
H7A	0.5162	0.6430	1.0674	0.058*	
C8	0.44261 (10)	0.8406 (3)	1.09868 (13)	0.0388 (4)	
C9	0.37639 (10)	0.7106 (3)	1.06543 (14)	0.0464 (5)	
H9A	0.3863	0.5977	1.0252	0.056*	
H9B	0.3626	0.6500	1.1207	0.056*	
C10	0.27588 (9)	0.7748 (3)	0.92530 (14)	0.0386 (4)	
C11	0.17921 (9)	0.9182 (3)	0.78351 (14)	0.0388 (4)	
C12	0.13539 (11)	1.1194 (3)	0.77934 (17)	0.0559 (6)	
H12A	0.1675	1.2379	0.7938	0.067*	
H12B	0.1069	1.1140	0.8272	0.067*	
C13	0.08603 (11)	1.1464 (3)	0.67914 (16)	0.0545 (6)	
H13A	0.0589	1.2764	0.6773	0.065*	
C14	0.03443 (11)	0.9647 (4)	0.65865 (17)	0.0556 (6)	
H14A	0.0029	0.9802	0.5953	0.067*	
H14B	0.0053	0.9610	0.7058	0.067*	
C15	0.07696 (12)	0.7641 (3)	0.66319 (17)	0.0580 (6)	
H15A	0.0437	0.6459	0.6497	0.070*	
C16	0.12637 (10)	0.7366 (3)	0.76323 (15)	0.0491 (5)	
H16A	0.0981	0.7328	0.8115	0.059*	
H16B	0.1523	0.6060	0.7661	0.059*	
C17	0.12141 (15)	0.7699 (4)	0.58855 (17)	0.0754 (8)	
H17A	0.0902	0.7830	0.5249	0.090*	
H17B	0.1483	0.6411	0.5908	0.090*	
C18	0.17292 (14)	0.9540 (5)	0.60795 (19)	0.0727 (8)	
H18A	0.2013	0.9586	0.5591	0.087*	
C19	0.22293 (11)	0.9277 (4)	0.70823 (17)	0.0599 (6)	
H19A	0.2506	0.8006	0.7101	0.072*	
H19B	0.2560	1.0442	0.7215	0.072*	
C20	0.13032 (13)	1.1555 (4)	0.6051 (2)	0.0729 (7)	
H20A	0.1629	1.2734	0.6181	0.087*	
H20B	0.0993	1.1740	0.5416	0.087*	
H1N2	0.2278 (11)	1.034 (4)	0.9084 (15)	0.051 (6)*	

### Atomic displacement parameters (Å^2^)

**Table:**

	*U*^11^	*U*^22^	*U*^33^	*U*^12^	*U*^13^	*U*^23^
S1	0.0513 (3)	0.0321 (3)	0.0554 (4)	0.0024 (2)	0.0028 (2)	−0.0036 (2)
N1	0.0388 (8)	0.0340 (9)	0.0481 (10)	0.0010 (6)	0.0057 (7)	−0.0023 (7)
N2	0.0455 (9)	0.0361 (11)	0.0575 (11)	0.0066 (7)	−0.0024 (8)	−0.0109 (8)
C1	0.0437 (11)	0.0419 (12)	0.0509 (12)	0.0046 (8)	0.0117 (9)	−0.0049 (8)
C2	0.0508 (12)	0.0453 (12)	0.0451 (12)	0.0021 (9)	0.0126 (9)	−0.0060 (9)
C3	0.0456 (11)	0.0434 (12)	0.0290 (10)	−0.0016 (8)	0.0082 (8)	0.0022 (7)
C4	0.0589 (12)	0.0463 (12)	0.0359 (11)	−0.0082 (9)	0.0067 (9)	−0.0005 (8)
C5	0.0464 (12)	0.0705 (16)	0.0440 (12)	−0.0172 (11)	0.0076 (10)	0.0007 (10)
C6	0.0420 (11)	0.0819 (17)	0.0419 (12)	0.0021 (11)	0.0121 (9)	0.0031 (11)
C7	0.0528 (12)	0.0557 (13)	0.0363 (11)	0.0108 (10)	0.0088 (9)	−0.0016 (9)
C8	0.0430 (10)	0.0406 (11)	0.0309 (10)	0.0017 (8)	0.0049 (8)	0.0020 (7)
C9	0.0493 (11)	0.0363 (11)	0.0490 (12)	0.0027 (9)	0.0020 (9)	0.0021 (8)
C10	0.0336 (9)	0.0353 (10)	0.0472 (12)	−0.0029 (7)	0.0101 (8)	0.0012 (8)
C11	0.0333 (9)	0.0306 (10)	0.0501 (12)	0.0003 (7)	0.0046 (8)	−0.0002 (8)
C12	0.0495 (12)	0.0392 (13)	0.0717 (15)	0.0064 (9)	−0.0010 (11)	−0.0092 (10)
C13	0.0521 (12)	0.0348 (12)	0.0698 (15)	0.0127 (9)	0.0002 (11)	0.0022 (9)
C14	0.0413 (11)	0.0607 (15)	0.0593 (14)	0.0029 (9)	0.0004 (10)	0.0047 (10)
C15	0.0582 (13)	0.0365 (13)	0.0661 (15)	−0.0082 (10)	−0.0124 (11)	0.0015 (10)
C16	0.0454 (11)	0.0386 (12)	0.0587 (13)	−0.0052 (9)	0.0024 (9)	0.0102 (9)
C17	0.1000 (19)	0.0659 (18)	0.0500 (15)	0.0366 (15)	−0.0034 (14)	−0.0084 (11)
C18	0.0681 (16)	0.098 (2)	0.0596 (16)	0.0250 (14)	0.0300 (13)	0.0246 (14)
C19	0.0428 (11)	0.0660 (16)	0.0751 (16)	0.0084 (10)	0.0219 (11)	0.0182 (12)
C20	0.0663 (15)	0.0633 (17)	0.0850 (18)	0.0017 (12)	0.0093 (14)	0.0356 (13)

### Geometric parameters (Å, º)

**Table:**

S1—C10	1.6906 (19)	C11—C16	1.526 (2)
N1—C10	1.352 (2)	C11—C12	1.533 (3)
N1—C1	1.473 (2)	C12—C13	1.532 (3)
N1—C9	1.477 (2)	C12—H12A	0.9700
N2—C10	1.353 (2)	C12—H12B	0.9700
N2—C11	1.482 (2)	C13—C20	1.503 (3)
N2—H1N2	0.85 (2)	C13—C14	1.512 (3)
C1—C2	1.517 (3)	C13—H13A	0.9800
C1—H1A	0.9700	C14—C15	1.516 (3)
C1—H1B	0.9700	C14—H14A	0.9700
C2—C3	1.501 (3)	C14—H14B	0.9700
C2—H2A	0.9700	C15—C17	1.511 (4)
C2—H2B	0.9700	C15—C16	1.531 (3)
C3—C4	1.385 (3)	C15—H15A	0.9800
C3—C8	1.389 (3)	C16—H16A	0.9700
C4—C5	1.387 (3)	C16—H16B	0.9700
C4—H4A	0.9300	C17—C18	1.522 (4)
C5—C6	1.373 (3)	C17—H17A	0.9700
C5—H5A	0.9300	C17—H17B	0.9700
C6—C7	1.385 (3)	C18—C20	1.524 (4)
C6—H6A	0.9300	C18—C19	1.538 (3)
C7—C8	1.387 (3)	C18—H18A	0.9800
C7—H7A	0.9300	C19—H19A	0.9700
C8—C9	1.501 (3)	C19—H19B	0.9700
C9—H9A	0.9700	C20—H20A	0.9700
C9—H9B	0.9700	C20—H20B	0.9700
C11—C19	1.510 (3)		
			
C10—N1—C1	121.12 (15)	C13—C12—H12A	109.6
C10—N1—C9	121.67 (16)	C11—C12—H12B	109.6
C1—N1—C9	117.13 (15)	C13—C12—H12B	109.6
C10—N2—C11	130.81 (17)	H12A—C12—H12B	108.1
C10—N2—H1N2	116.1 (15)	C20—C13—C14	110.2 (2)
C11—N2—H1N2	111.1 (15)	C20—C13—C12	109.55 (18)
N1—C1—C2	112.39 (16)	C14—C13—C12	109.20 (18)
N1—C1—H1A	109.1	C20—C13—H13A	109.3
C2—C1—H1A	109.1	C14—C13—H13A	109.3
N1—C1—H1B	109.1	C12—C13—H13A	109.3
C2—C1—H1B	109.1	C13—C14—C15	108.90 (17)
H1A—C1—H1B	107.9	C13—C14—H14A	109.9
C3—C2—C1	110.89 (16)	C15—C14—H14A	109.9
C3—C2—H2A	109.5	C13—C14—H14B	109.9
C1—C2—H2A	109.5	C15—C14—H14B	109.9
C3—C2—H2B	109.5	H14A—C14—H14B	108.3
C1—C2—H2B	109.5	C17—C15—C14	109.51 (19)
H2A—C2—H2B	108.0	C17—C15—C16	109.45 (18)
C4—C3—C8	120.13 (18)	C14—C15—C16	110.37 (19)
C4—C3—C2	124.29 (18)	C17—C15—H15A	109.2
C8—C3—C2	115.58 (17)	C14—C15—H15A	109.2
C3—C4—C5	119.7 (2)	C16—C15—H15A	109.2
C3—C4—H4A	120.1	C11—C16—C15	109.25 (16)
C5—C4—H4A	120.1	C11—C16—H16A	109.8
C6—C5—C4	120.06 (19)	C15—C16—H16A	109.8
C6—C5—H5A	120.0	C11—C16—H16B	109.8
C4—C5—H5A	120.0	C15—C16—H16B	109.8
C5—C6—C7	120.60 (19)	H16A—C16—H16B	108.3
C5—C6—H6A	119.7	C15—C17—C18	109.80 (19)
C7—C6—H6A	119.7	C15—C17—H17A	109.7
C6—C7—C8	119.6 (2)	C18—C17—H17A	109.7
C6—C7—H7A	120.2	C15—C17—H17B	109.7
C8—C7—H7A	120.2	C18—C17—H17B	109.7
C7—C8—C3	119.76 (18)	H17A—C17—H17B	108.2
C7—C8—C9	122.91 (18)	C17—C18—C20	109.3 (2)
C3—C8—C9	117.32 (16)	C17—C18—C19	108.9 (2)
N1—C9—C8	110.52 (16)	C20—C18—C19	109.4 (2)
N1—C9—H9A	109.5	C17—C18—H18A	109.7
C8—C9—H9A	109.5	C20—C18—H18A	109.7
N1—C9—H9B	109.5	C19—C18—H18A	109.7
C8—C9—H9B	109.5	C11—C19—C18	109.80 (17)
H9A—C9—H9B	108.1	C11—C19—H19A	109.7
N1—C10—N2	114.44 (17)	C18—C19—H19A	109.7
N1—C10—S1	122.50 (14)	C11—C19—H19B	109.7
N2—C10—S1	123.05 (15)	C18—C19—H19B	109.7
N2—C11—C19	111.34 (16)	H19A—C19—H19B	108.2
N2—C11—C16	113.34 (16)	C13—C20—C18	109.52 (18)
C19—C11—C16	110.43 (18)	C13—C20—H20A	109.8
N2—C11—C12	104.80 (16)	C18—C20—H20A	109.8
C19—C11—C12	109.13 (17)	C13—C20—H20B	109.8
C16—C11—C12	107.50 (16)	C18—C20—H20B	109.8
C11—C12—C13	110.23 (17)	H20A—C20—H20B	108.2
C11—C12—H12A	109.6		
			
C10—N1—C1—C2	−178.44 (16)	C10—N2—C11—C12	−179.4 (2)
C9—N1—C1—C2	−1.5 (2)	N2—C11—C12—C13	178.19 (16)
N1—C1—C2—C3	47.4 (2)	C19—C11—C12—C13	58.8 (2)
C1—C2—C3—C4	131.5 (2)	C16—C11—C12—C13	−60.9 (2)
C1—C2—C3—C8	−48.2 (2)	C11—C12—C13—C20	−59.5 (2)
C8—C3—C4—C5	−2.4 (3)	C11—C12—C13—C14	61.2 (2)
C2—C3—C4—C5	177.96 (19)	C20—C13—C14—C15	60.7 (2)
C3—C4—C5—C6	0.2 (3)	C12—C13—C14—C15	−59.6 (2)
C4—C5—C6—C7	1.7 (3)	C13—C14—C15—C17	−60.3 (2)
C5—C6—C7—C8	−1.4 (3)	C13—C14—C15—C16	60.2 (2)
C6—C7—C8—C3	−0.8 (3)	N2—C11—C16—C15	175.54 (16)
C6—C7—C8—C9	−179.65 (18)	C19—C11—C16—C15	−58.8 (2)
C4—C3—C8—C7	2.7 (3)	C12—C11—C16—C15	60.2 (2)
C2—C3—C8—C7	−177.63 (17)	C17—C15—C16—C11	59.4 (2)
C4—C3—C8—C9	−178.40 (17)	C14—C15—C16—C11	−61.2 (2)
C2—C3—C8—C9	1.2 (3)	C14—C15—C17—C18	60.0 (2)
C10—N1—C9—C8	133.07 (17)	C16—C15—C17—C18	−61.1 (2)
C1—N1—C9—C8	−43.8 (2)	C15—C17—C18—C20	−58.9 (3)
C7—C8—C9—N1	−136.37 (18)	C15—C17—C18—C19	60.6 (2)
C3—C8—C9—N1	44.8 (2)	N2—C11—C19—C18	−174.24 (19)
C1—N1—C10—N2	−0.3 (3)	C16—C11—C19—C18	58.9 (2)
C9—N1—C10—N2	−177.09 (16)	C12—C11—C19—C18	−59.0 (2)
C1—N1—C10—S1	−179.06 (14)	C17—C18—C19—C11	−59.3 (3)
C9—N1—C10—S1	4.2 (2)	C20—C18—C19—C11	60.2 (3)
C11—N2—C10—N1	169.16 (18)	C14—C13—C20—C18	−60.1 (3)
C11—N2—C10—S1	−12.1 (3)	C12—C13—C20—C18	60.0 (3)
C10—N2—C11—C19	−61.5 (3)	C17—C18—C20—C13	58.8 (3)
C10—N2—C11—C16	63.7 (3)	C19—C18—C20—C13	−60.4 (3)

### Hydrogen-bond geometry (Å, º)

**Table:**

*D*—H···*A*	*D*—H	H···*A*	*D*···*A*	*D*—H···*A*
C16—H16*B*···S1	0.97	2.72	3.365 (2)	125
